# Association between retinal vessel density and postoperative time after primary repair of rhegmatogenous retinal detachment

**DOI:** 10.1371/journal.pone.0258126

**Published:** 2021-10-01

**Authors:** Miklós D. Resch, Anikó Balogh, Gábor Lászik, Zoltán Z. Nagy, András Papp

**Affiliations:** 1 Department of Ophthalmology, Semmelweis University, Budapest, Hungary; 2 Department of Ophthalmology, Uzsoki Hospital, Budapest, Hungary; Medizinische Universitat Graz, AUSTRIA

## Abstract

The study aimed at a quantitative evaluation of macular vasculature after primary repair of rhegmatogenous retinal detachment (RRD) in correlation with the elapsed postoperative time. Optical coherence tomography angiography (OCT-A) was performed in 66 eyes of 33 patients in a retrospective case-control study: superficial and deep retinal vessel density (VD) of the whole image, fovea, parafovea, non-flow area, and foveal avascular zone (FAZ) were measured. Data of eyes with RRD were compared to the healthy fellow eyes in 3 groups according to the elapsed time after surgery: RD1: 6–12 months (n = 10), RD2: 1–2 years (n = 10), and RD3: 2–10 years (n = 13). In RD1 VD was significantly lower in the superficial parafoveal, deep parafoveal, and deep whole area compared to the fellow eyes. In RD3 VD was significantly lower in the superficial fovea, parafovea, whole image, and deep fovea, the non-flow area was significantly enlarged. OCT-A demonstrated a significant reduction in the superficial and deep regions of the macular vasculature after the repair of RRD. The deep area is more affected in the early postoperative period and the superficial region and the extent of the non-flow area are more involved after a longer postoperative time.

## Introduction

Rhegmatogenous retinal detachment (RRD) is the separation of the neurosensory retina from retinal pigment epithelium caused by liquified vitreous accumulation through a retinal break. The treatment of this condition is surgical, including pars plana vitrectomy (PPV) with gas or silicone oil endotamponade, scleral buckling, a combination of the two, and pneumatic retinopexy. Despite successful surgery resulting in reattached retina visual acuity remains impaired in almost 40% of cases, especially when the macula was detached or proliferative vitreoretinopathy developed after surgery [1]. The cause of suboptimal visual recovery after successful surgery is an extensively studied problem. A lot of advanced techniques have been used to investigate the etiology of poorer visual outcomes: microperimetry [2], intravitreal cytokine profile [3], spectral-domain optical coherence tomography (SD-OCT) [4], OCT-angiography (OCT-A) imaging. Kobayashi et al retrospectively analyzed SD-OCT images of eyes with successfully reattached macula-off RRD. They found that the thickness of the ellipsoid zone—pigment epithelium and cone density increased during foveal regeneration [5]. Park et al studied the pre-and postoperative factors associated with visual outcome after macula-off RRD surgery and they found that postoperatively predictive factors on SD-OCT were the outer retinal microstructure, especially the photoreceptor outer segment layer [6].

Even if the microstructure of the retina seems repaired and there is not any abnormality on conventional OCT image patients might have functional deterioration such as color vision defects and persistent metamorphopsia [7]. With the help of OCT-A a depth-resolved (superficial and deep layer) retinochoroidal microvasculature becomes visible in a noninvasive way by using endoluminal flow as an intrinsic contrast. By performing a quantitative analysis of macular vessel density (VD) on OCT-A images, it made it possible to examine the mechanisms that are potentially responsible for the poorer prognosis in cases of RRD. Recently some OCT-A studies [8–11] were published on the relatively short term (up to 12 months) effect of retinal detachment surgery on the macular vasculature.

The purpose of this study was to analyze the quantitative characteristics of the macular superficial and deep VD after a very long follow-up time (from 6 months to 10 years) following primary repair of RRD and to analyze the association between elapsed postoperative time and vascular changes.

## Material and methods

The present study was approved by the Regional and Institutional Committee of Science and Research Ethics (SE TUKEB No. 253/2016.) and performed following the tenets of the Declaration of Helsinki and applicable national and local requirements. All patients signed written informed consent before entering the study.

### Participants

Sixty-six eyes of 33 patients, who underwent successful primary repair of RRD at Semmelweis University Department of Ophthalmology between 2009 and 2019, were studied retrospectively. Patients with RRD in one eye only were included in the study, the healthy fellow eyes without the history of RRD served as controls. Patients who had a history of previous vitreoretinal surgery, penetrating injury, uveitis, aphakia, age-related macular degeneration, macular hole, proliferative vitreoretinopathy, diabetic retinopathy, and uncontrolled glaucoma were excluded. Eyes with a single surgery were included, only cataract surgery and silicone oil explantation were allowed as a further intervention. If silicone oil was applied, it was removed at the timepoint of examination. Previous retinal detachment intervention or retinal break treatment in the fellow eye were exclusive. Eyes with an axial length of more than 26 mm were excluded. Non-ocular exclusion criteria were any type of diabetes, uncontrolled hypertension, and systemic use of antiangiogenic drugs.

Eyes with RRD were compared with healthy fellow eyes and were divided into three groups according to the elapsed time after primary repair: RD1: six months to one year (n = 10), RD2: one year to two years (n = 10), and RD3: more than two (2 to 10) years (n = 13). In RD3 follow-up period varied from 25 to 126 months postoperatively to investigate the very long-term effects of RD surgery. Patients demographics and clinical data are summarized in **Tables 1** and **2.**

**Table 1:** Demographic and clinical data of patients.

	RD1	RD2	RD3
N	10	10	13
Male/Female	7/3	4/6	7/6
Age (year)	53.0 ± 14.2	53.4 ± 18.26	57.3± 17.95
BCVA (decimal)	0.67 ± 0.31	0.58 ± 0.38	0.63 ± 0.38
Axial length (mm)	22.7 ± 3.1	22.4 ± 2.3	22.9 ± 2.8
Symptom duration (day)	15.7 ± 20.1	7.4 ± 9.3	9.3 ± 8.7

No statistical difference was found in age among groups

**Table 2:** Retinal detachment characteristics.

	Extent of RD (quadrants)	Macula on/off (n)	Location of tear (%)				Type of surgery (n)			Endotamp.
			superior	inferior	temporal	nasal	PPV	SB	PPV+SB	SIO/C3F8
RD1	2.25 ± 0.63	5/5	40	40	10	10	10	0	0	0/10
RD2	1.9 ± 0.57	9/1	50	30	0	20	7	2	1	2/6
RD3	2.23 ± 0.93	6/7	54	23	23	0	10	2	1	5/6

*Statistical difference was found in symptom duration and macula on/off ratio.

RD: retinal detachment, PPV: pars plana vitrectomy; SB: scleral buckle; n: number, BCVA: best corrected visual acuity, SIO: Silicone oil. BCVA was set at the time of OCT-A image acquisition

### Examinations, image acquisition, and analysis

All subjects underwent a comprehensive ophthalmic examination, including best-corrected visual acuity (BCVA) assessment (ETDRS Charts), slit lamp, and fundus examinations. After pupil dilation, OCT-A was performed, using the AngioVue OCT-A system (RTVue-XR Avanti, OptoVue, Fremont, CA, USA). Scans with the highest resolution were obtained in the central 3×3 mm area, centered on the foveola. Scans with insufficient image quality (lower than 5/10 determined by the inbuilt imaging software) were excluded [12]. The superficial capillary plexus (SCP) was detected automatically between the internal limiting membrane (ILM) and the inner plexiform layer (IPL); and the deep capillary plexus (DCP) between the IPL and the outer plexiform layer (OPL). Superficial and deep VD was evaluated in the whole image, in the central 1 mm area (fovea), and the 3 mm ring-shaped parafoveal area. Superficial non-flow area and foveal avascular zone (FAZ) areas were measured, using the built-in AngioAnalytics software (Version ReVue 2018.0.0.18) OptoVue system with an automated segmentation algorithm. The axial length of the eyes was measured with the IOL Master 500 (Zeiss, Carl Zeiss Meditec AG, Jena, Germany).

### Statistical analysis

Statistical analysis was performed via dedicated statistical software GraphPad Prism (GraphPad Software Inc, La Jolla, CA) version 5.0. Shapiro Wilk test was applied for testing normal disribution of the data, which revealed non-normal distribution (p>0.05). Wilcoxon matched-pairs signed rank test was used to assess the differences between RD and corresponding fellow eyes (for all patients and in three groups) in the superficial fovea, superficial parafovea, superficial whole image, deep fovea, deep parafovea, deep whole image, non-flow, and FAZ area. We performed a subgroup analysis to examine the differences in VD between macula-on and macula-off RD, and silicon oil and C3F8 gas endotamponade. Spearman-rank correlation was calculated for the VD values and postoperative time. The quantitative data were expressed in median along with range (min-max). P-values <0.05 were set to indicate statistically significant results.

## Results

### Clinical data

A total of 33 patients (18 men, 15 women) with a mean age of 58.8 ± 16.6 (18 to 85) years were included in this study. The patients’ demographics and clinical data of the three groups are summarized in **Tables 1** and **2.** The difference in age between the groups was not statistically significant.

### OCT-A image analysis

Quantitative OCT-A results are summarized for all eyes in **Table 3** and for groups in **Table 4.**

**Table 3:** Results of OCT-A measurements for all eyes.

	RD	Control	p (Wilcoxon)
SPF fovea	18.20 (5.1–35.8)	2.26 (6.5–33.2)	0.1555
SPF parafovea	40.35 (24.4–51.4)	47.60 (3.9–56.8)	0.0002***
SPF whole	38.80 (23.5–49.8)	44.70 (30.7–53.3)	0.0012**
D fovea	31.90 (20.6–50.7)	36.00 (15.8–47.8)	0.0121*
D parafovea	48.05 (32.4–66.2)	52.40 (43.5–60.5)	0.0113*
D whole	45.70 (30.6–62.0)	50.60 (42.2–57.3)	0.0058**
Non-flow	0.444 (0.170–1.606)	0.415 (0.202–0.728)	0.221
FAZ	0.216 (0.052–0.507)	0.227 (0.064–0.542)	0.3694

Superficial (SPF) and deep (D) vascular density in the fovea, parafovea, and the whole image. Non-flow area and foveal avascular zone (FAZ). Data are shown as median and mean (min-max). Statistically significant differences between the groups are marked by an asterisk.

* p<0.05

** p<0.01

*** p<0.001.

**Table 4:** Results of OCT-A measurements in groups.

	RD1	Control 1	RD2	Control 2	RD3	Control 3
**SPF fovea**	22.55	22.05	18.15	19.05	17.10	23.20*
	8.7–30.2	13.2–32.6	6.7–35.8	6.5–33.2	5.1–33.1	10.0–32.4
**SPF parafovea**	46.00	51.90*	41.10	46.70	38.90	47.60*
	24.4–51.4	44.7–52.8	30.6–46.8	33.9–56.8	25.2–51.3	36.4–54.5
**SPF whole**	43.85	45.70	38.90	44.35	35.40	45.6*
	24.4–49.0	35.4–50.5	29.2–44.4	30.7–53.3	23.5–49.8	34.9–51.0
**D fovea**	34.30	39.90	32.56	33.90	30.80	36.90*
	20.8–43.6	31.1–46.8	20.6–43.8	15.8–44.7	20.7–50.7	24.3–47.8
**D parafovea**	48.05	52.35*	50.40	53.40	47.30	52.30
	43.9–53.9	44.6–58.8	37.4–62.4	43.5–55.7	32.4–66.2	44.4–60.5
**D whole**	46.65	50.45*	46.40	50.85	45.70	48.80
	40.8–51.4	42.2–56.0	30.6–58.6	42.6–53.0	33.0–62.0	43.4–57.3
**Non-flow**	0.420	0.435	0.430	0.445	0.470	0.384*
	0.253–0.729	0.202–0.590	0.170–0.714	0.295–0.728	0.193–1.606	0.214–0.641
**FAZ**	0.191	0.205	0.212	0.225	0.223	0.238
	0.095–0.366	0.086–0.342	0.052–0.507	0.066–0.542	0.055–0.440	0.064–0.380

Superficial (SPF) and deep (D) vascular density in the fovea, parafovea, and the whole image. Non-flow area and foveal avascular zone (FAZ). Data are shown as median and range (min-max). Statistically significant differences between the groups are marked by an asterisk.

* p<0.05.

#### Superficial retinal VD

**Fig 1:**
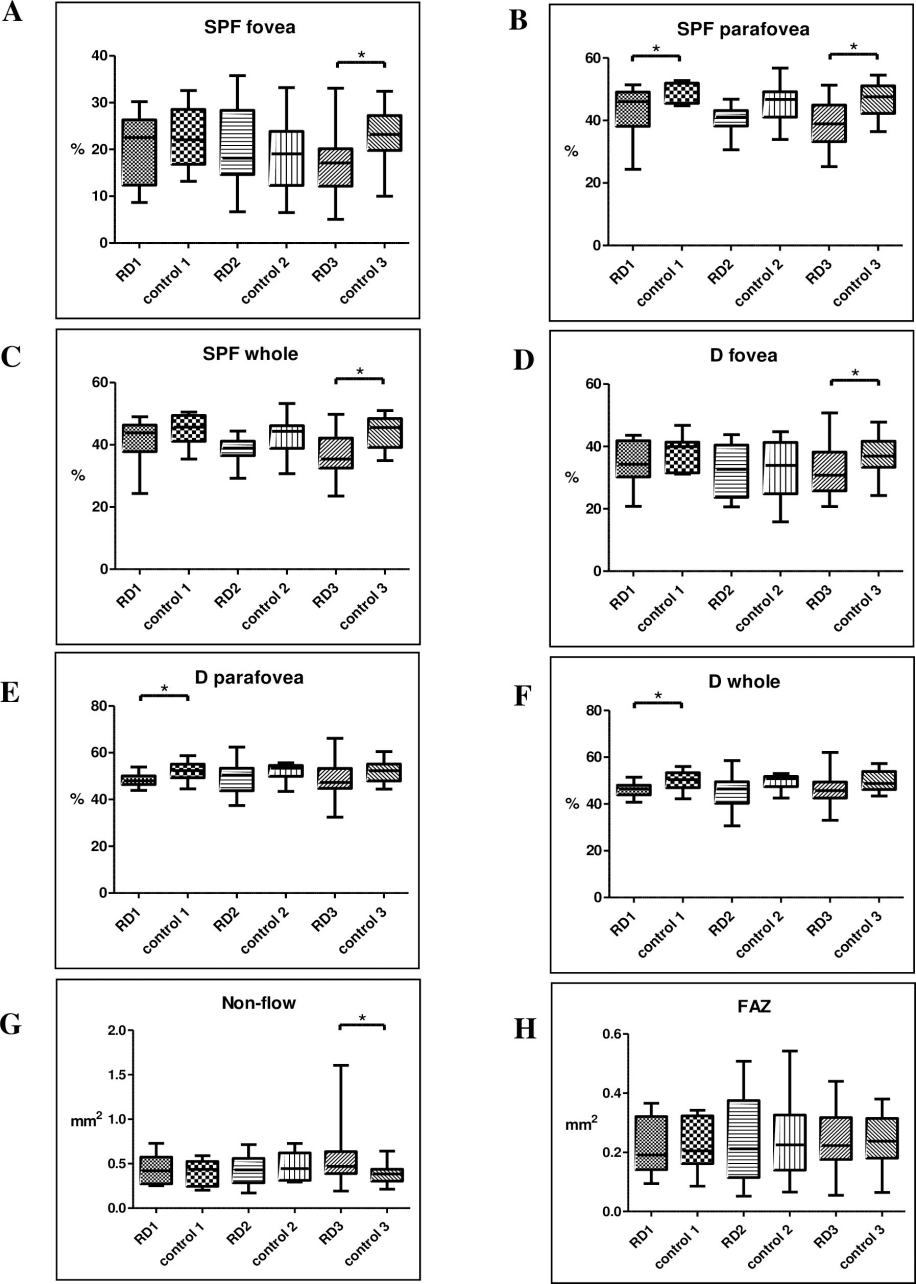
Diagram of the mean and SD of superficial (SPF, A-C) and deep retinal vascular density (D, D-F), non-flow area (G), and FAZ (foveal avascular zone, H) in all groups. Statistically significant differences between the groups are marked by an asterisk. * p<0.05.

The mean superficial VD for all patients was not different in the fovea but was lower in the parafoveal region and the whole image compared to that of in controls (**Table 3**). The density of superficial retinal vasculature in the fovea however was significantly lower in RD3 compared to the fellow eye (17.39 ± 7.26% and 22.58 ± 6.63%), while we could not find a difference in RD1 and RD2 (**Fig 1A**). Parafoveal VD was lower in RD1 (43.04 ± 8.14% and 49.94 ± 3.38%) and RD3 (38.20 ± 7.40% and 46.73 ± 5.45%) (**Fig 1B**). The VD in the whole image was significantly lower in RD3 (36.57 ± 6.81% and 44.12 ± 5.34%), whereas there was no significant difference in the first two groups (**Fig 1C**).

#### Deep retinal VD

Regrading all eyes, the mean VD in the deep plexus was lower in the RD eyes compared to that of controls in all regions (fovea, parafovea, whole image) (**Table 3**). Deep retinal VD in the fovea was lower in RD3 compared to fellow eyes (32.30 ± 8.20% and 37.32 ± 6.52%) (**Fig 1D**). Deep parafoveal region (48.20 ± 2.83% and 52.16 ± 4.02%) and whole area (46.03 ± 2.94% and 50.03% ± 4.18%) were significantly lower in RD 1 compared to controls, while we could not find a difference in RD2 and RD3 (**Fig 1E and 1F**).

#### Non-flow area and FAZ

In general, no difference was found in the non-flow area and FAZ between RD and fellow eyes (**Table 3**). The non-flow area was significantly higher only in RD3 compared to the fellow eye (0.57mm2 ± 0.34mm2 and 0.398mm2 ± 0.127mm2) (**Fig 1G**). We could not find a significant difference in FAZ in any of the groups (**Fig 1H**).

#### Correlation of VD with postoperative time

**Fig 2:**
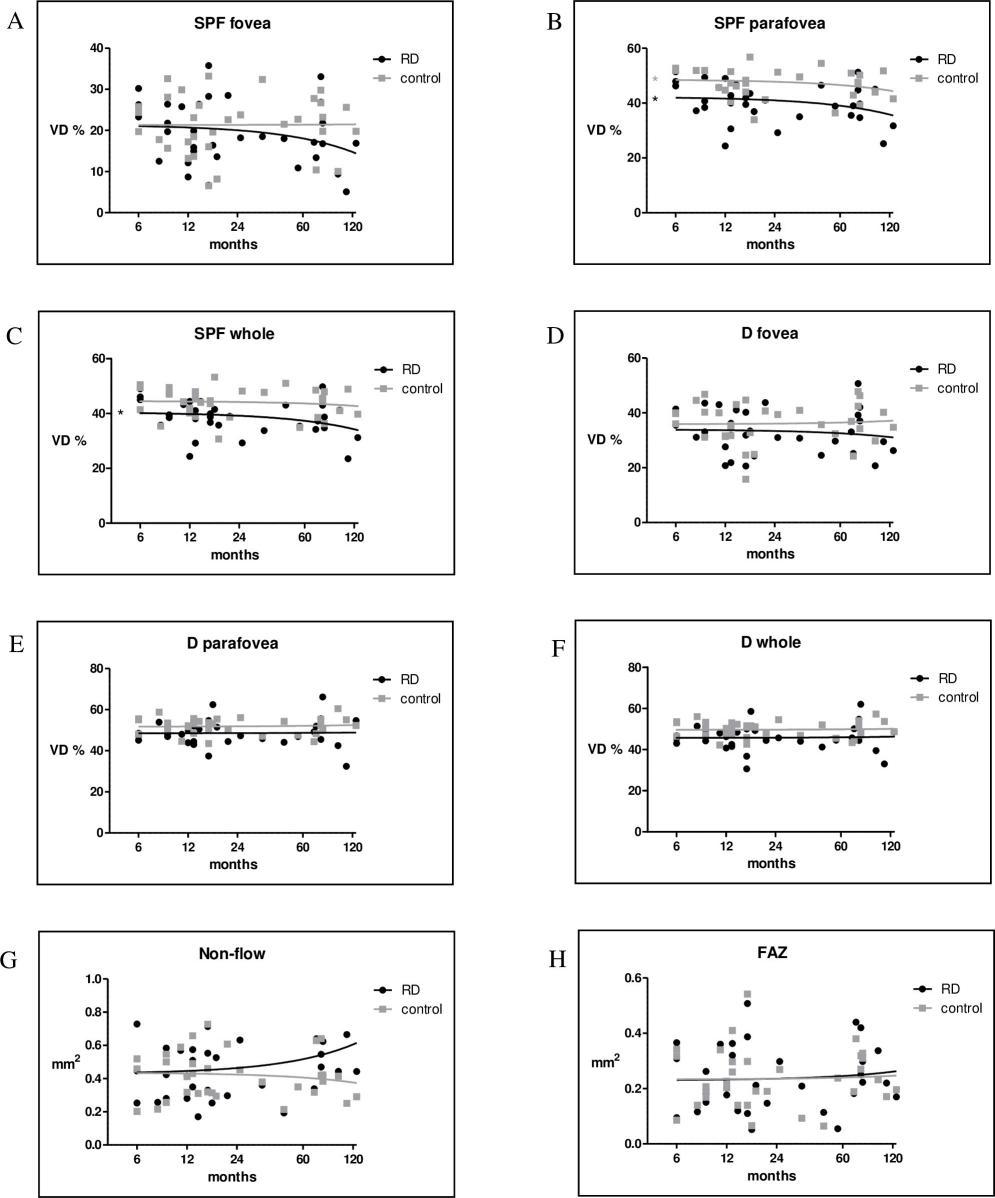
Correlation diagram between time and superficial (SPF, A-C), deep (D) retinal vascular density (D-F), non-flow area (G), and FAZ (foveal avascular zone, H). VD: vessel density. Statistically significant differences between the groups are marked by an asterisk. * p<0.05.

Spearman rank correlation demonstrated a significant correlation between elapsed postoperative time and VD in the superficial parafovea area of the RD (p = 0,048, r = -0.352) and control eye (p = 0,0161, r = -0.416) as well, but no correlation was found in the superficial fovea (**Fig 2A and 2B**). In the superficial whole area, VD was significantly correlated with time only in the eyes with RD (p = 0,0253, r = -0.389), while we could not find a similar correlation in the control group (**Fig 2C**). Macular VD was lower in the RD eyes compared to controls in the superficial and deep area, but superficial VD difference is more pronounced (**Fig 2A–2F**). We could not find correlation between time and the non-flow, and FAZ area, but in the non-flow area of RD eyes, there is a growth with the elapsed time after surgery (**Fig 2G**).

**Fig 3:**
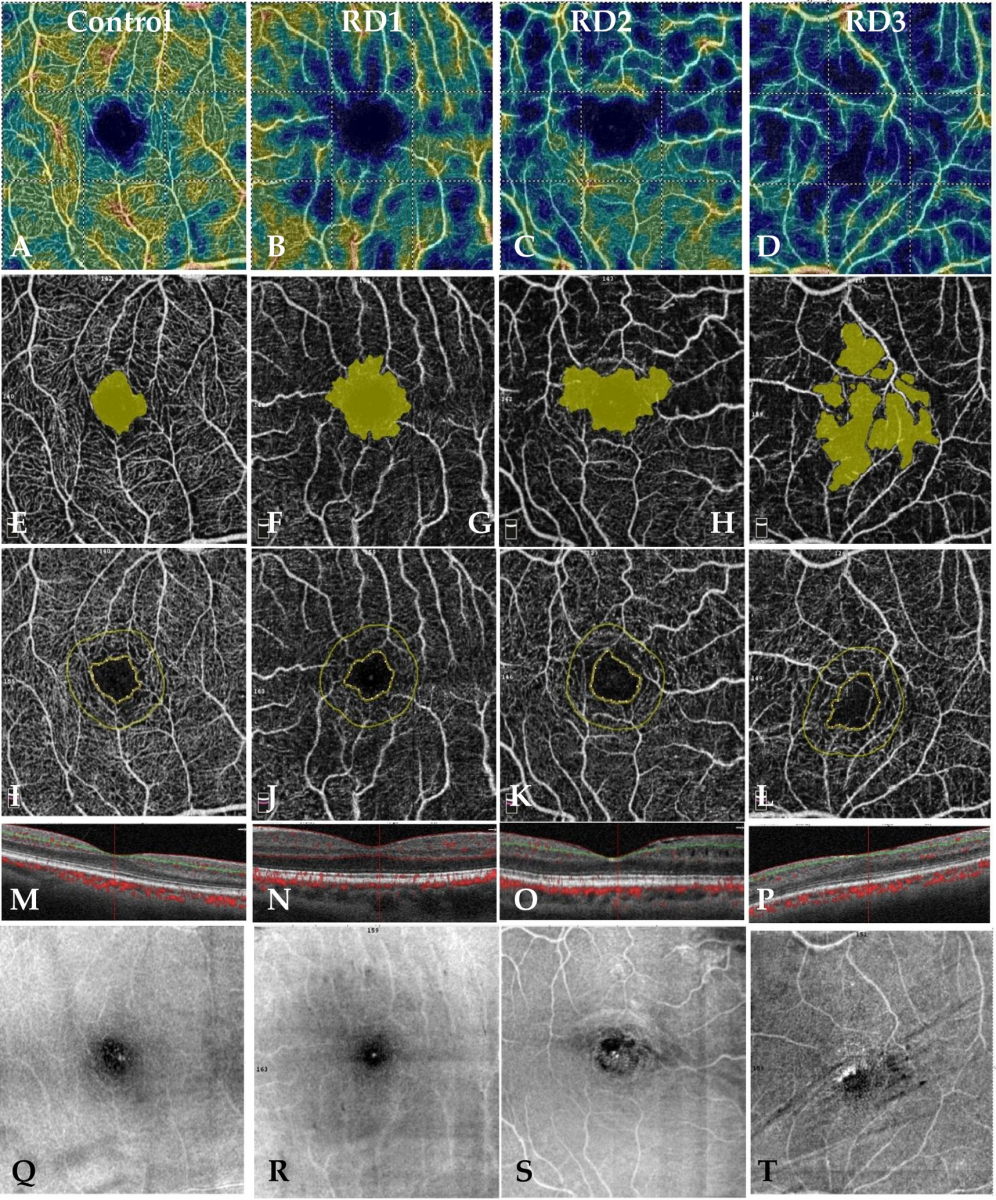
Demonstrative OCT-A images from all groups. VD—Vessel density (A-D). Non-flow area (E-H), FAZ—Foveal avascular zone (I-L), OCT image (M-P), and en face image (Q-T).

**Fig 3** shows representative images of patients from control groups and RD1, RD2, and RD3 groups. The vessel density decrease and the increased FAZ and non-flow area are characteristic for eyes after retinal detachment surgery.

#### Subgroup analysis

**Fig 4:**
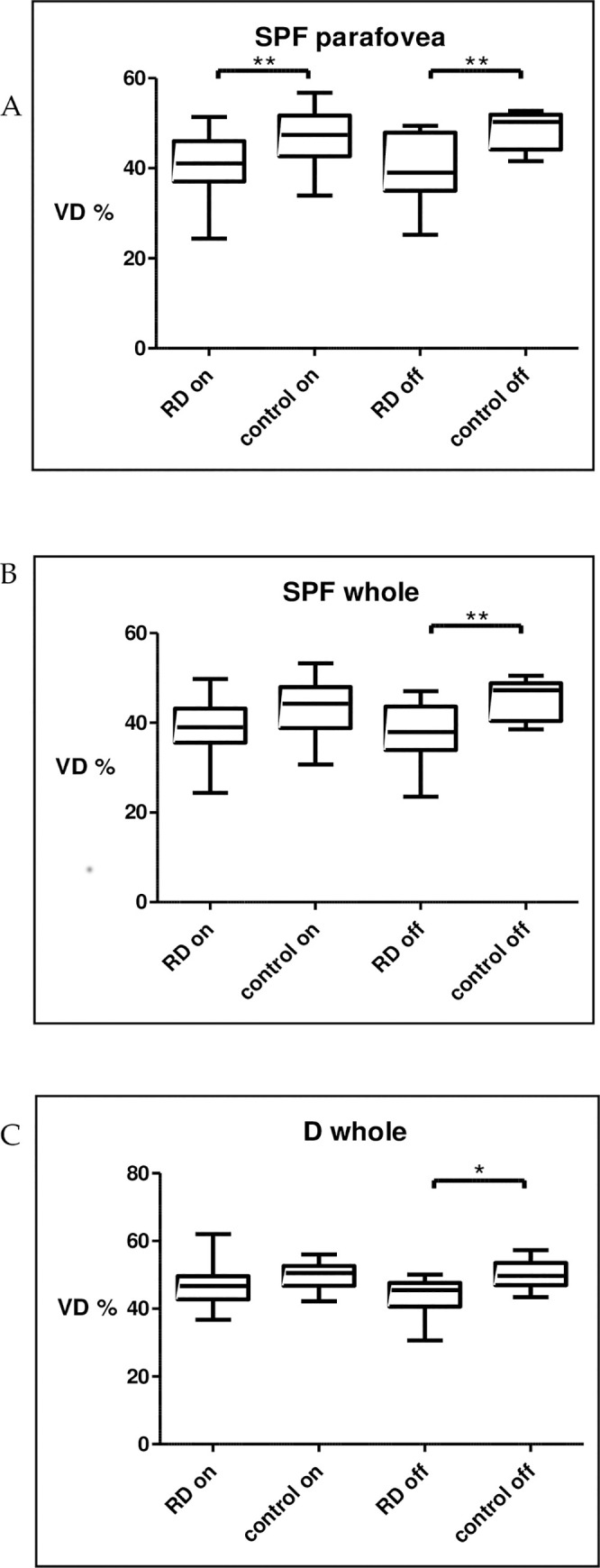
Significant differences in macula on-off subgroups. Superficial VD in parafovea (A.) and whole image (B.). C. Deep VD in whole image.

In the subgroup analysis between macula-on (n = 21) and macula-off (n = 12) RD, and silicon oil (n = 7) and C3F8 gas (n = 22) endotamponade Wilcoxon matched-pairs signed rank test was used to assess the differences. We found that the VD of macula-off eyes was significantly lower compared to controls in the superficial whole (p = 0.0049) and deep whole area (p = 0.0342), while we could not find a difference between macula-on eyes and controls. The VD of the superficial parafoveal region was significantly lower in both macula-on (p = 0.0085) and macula-off eyes (p = 0.0098) compared to the fellow eyes (**Fig 4**). In the other areas, we did not find a significant difference according to the macular status.

**Fig 5:**
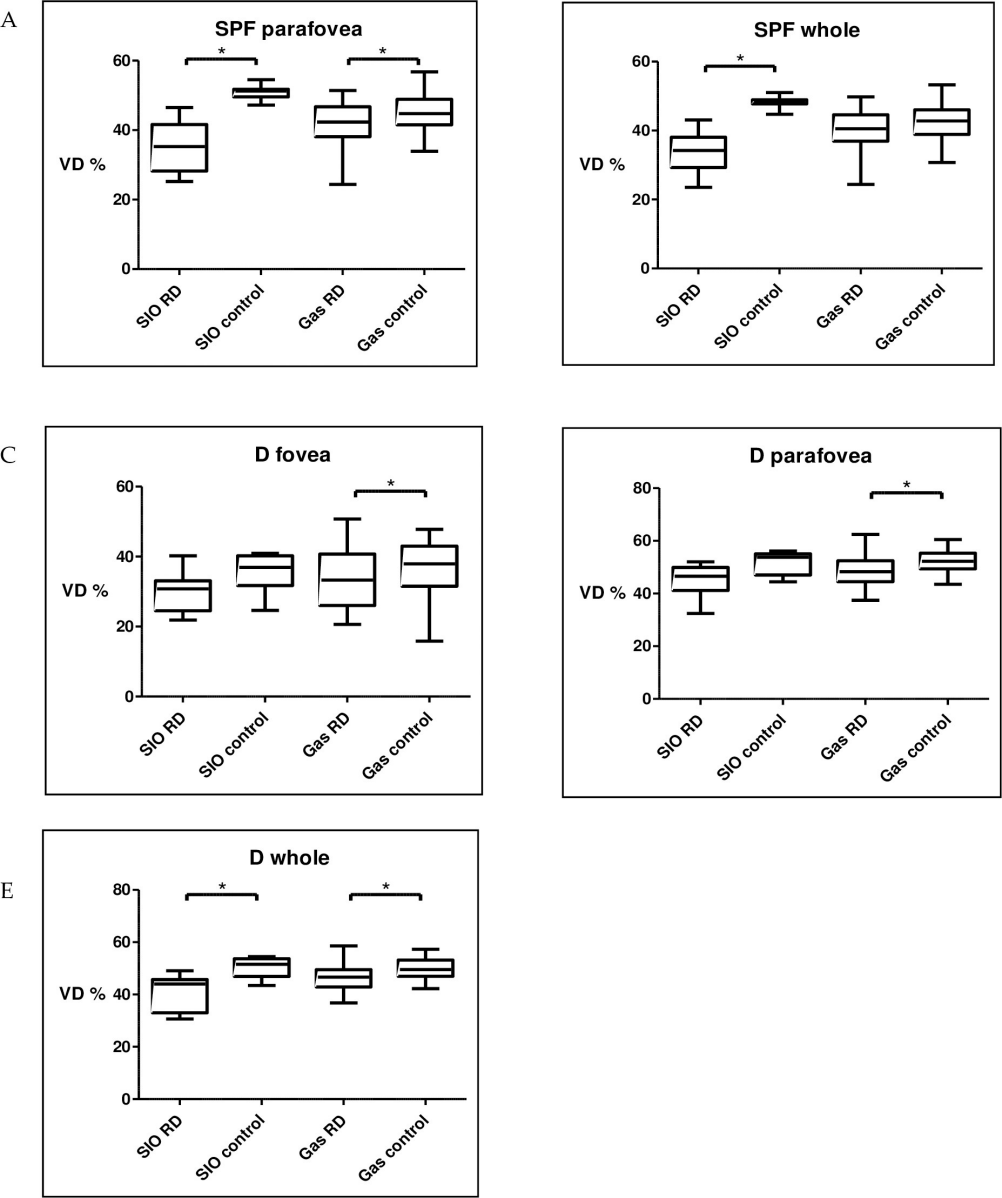
Significant differences according to endotamponade in SIO and gas subgroups. Superficial VD in parafovea (A.) and whole image (B.). Deep VD in fovea (C.), prafovea (D.) and whole image (E.).

The VD of eyes with silicone oil endotamponade was significantly lower compared to fellow eyes in the superficial whole area (p = 0.0223). Eyes with C3F8 endotamponade had a lower VD in the deep foveal (p = 0.0424) and deep parafoveal region (p = 0.0273). The VD was significantly lower in superficial parafovea (SIO: p = 0.0313; C3F8: p = 0.0438) and deep whole area (SIO: p = 0.0469; C3F8: p = 0.0377) in both of the silicon oil and C3F8 group (**Fig 5**).

## Discussion

Our results demonstrate, that significant vascular damage (decreased VD) of the macula can be detected by OCT-A even several years after successful RRD surgery. Before OCT-A imaging was available for clinicians to examine patients with various retinal diseases, morphological changes could be analyzed only with conventional OCT. Many researchers studied retinal microstructure after RRD to explore the causes of poor functional outcome after surgical repair. Karacorlu et al. evaluated the prognostic factors of recent onset macula-off RRD, epiretinal membrane formation was found as the only factor affecting postoperative visual acuity [13]. Sato et al. found that retinal thickness in the temporal parafoveal subfield may most closely reflect postoperative BCVA after macula-off RRD repair [14]. Han et al. analyzed the retinal layers with automated segmentation in the macular region and they found that outer nuclear layer thickness can be used as a potential biomarker to predict visual outcome after RRD repair [15].

With the invention of OCT-A, the microvasculature of the retina became visible that expands our knowledge of the morphological changes after RRD. The first OCT-A devices had no eye-tracking function which caused a lot of segmentation errors, motion artifacts, and it encumbered to make a good quality image, especially from eyes with poor function [16]. After eye-tracking software was evolved image quality emerged. Lauerman et al evaluated the impact of eye-tracking technology on OCT-A image quality and the manifestation of motion artifacts, and they concluded that active eye-tracking technology offers an improved image quality at the expense of higher acquisition time [17].

In the first OCT-A studies only the FAZ area could be measured in the images. Woo et al. retrospectively analyzed 34 eyes with macula-off RRD after successful PPV. They found that both superficial and deep FAZ areas were significantly larger in the macula-off group than in the macula-on group following surgery, while in our study this difference was not detectable. They concluded that the enlargement of FAZ indicates that there is ischemic damage to the retinal capillary plexus in the fovea [8].

As the software improved, initially the quantitative analysis of superficial VD became possible and the deep VD followed afterward. Wang et al retrospectively studied 14 eyes with macula-off RRD after PPV throughout postoperative 12 weeks in the SCP, DCP, and choriocapillary plexus (CCP). They found a significant increase in SCP flow density, as well as the DCP flow density and the CCP flow density over time [9]. Tsen et al. evaluated the changes in the microcirculation of retinal layers and choroid following successful repair of macula-off RRD of 28 eyes. They concluded RD eyes had a significantly lower VD than fellow eyes after surgery, and the combined procedure might result in a lower VD, as compared with a scleral buckle or PPV alone [10]. Tsen et al compared the different types of surgical approaches, but they did not examine the impact of the endotamponade on the VD. Lee et al. examined thirty-eight patients with unilateral RRD treated with vitrectomy and silicone oil endotamponade. They found that FAZ area in the DCP was larger and the VD in DCP was lower compared to the contralateral eyes [18]. We also examined the VD changes in eyes filled with silicone oil, and additionally compared them to eyes filled with C3F8. We found that VD eyes in the superficial whole area after silicone oil endotamponade was significantly lower compared to fellow eyes. Eyes with C3F8 endotamponade had a lower VD in the deep foveal and deep parafoveal region. The VD was significantly lower in superficial parafovea and deep whole area in both of the silicone oil and C3F8 group. Agarwal et al. also examined 19 patients after macula-off RRD reattachment surgery and compared them to healthy subjects with no ocular pathologies. At three months postoperatively they found a significant reduction in mean capillary density index, fractal dimension, and vascular perfusion, and branching pattern in patients after surgery for RRD [11]. Ng et al. associated the change in the FAZ and VD with final BCVA in eyes after macula-off RRD surgery, and investigated the evolution of FAZ and VD during 12 months of follow-up. At 12 months postoperatively, FAZ difference of the DCP and BCVA difference were correlated, and there was no evidence that FAZ and VD changed during follow-up [19].

Bonfiglio et al. studied the patients’ eyes after macula-on and macula-off RRD repair with OCT-A. They did not find a difference in foveal macular thickness and FAZ area compared to fellow eyes. In macula-on RRD eyes, a lower parafoveal deep VD was detected. In macula-off RRD eyes, lower superficial parafoveal VD and deep foveal and parafoveal VD were observed. BCVA was related to the FAZ area, foveal superficial VD, and parafoveal deep VD [20]. Our results are partly consistent with Bonfiglio’s, FAZ area was not different in macula-on and macula-off eyes compared to fellow eyes, and superficial parafoveal VD was significantly lower in macula-off and macula-on eyes. Beyond these results, we found that the VD of superficial and deep whole areas are significantly lower in macula-off eyes compared to controls. Hong et al. besides analysis of VD in eyes after RRD surgery, compared retinal and choroidal VD and retinal layer thickness between eyes with macula-on and macula-off RRD at six months postoperatively. They concluded that the CCP VD could be related to the anatomical restoration of the outer retinal layer in macula-off RRD. [20]. Jiang et al performed a prospective evaluation of RRD patients after silicone implantation and found dynamic increase of the VD in SCP and DCP as well up to week 12, when it started to decrease [21]. Fang et al compared air and silicone oil endotamponade, and observed decrease of VD in both groups [22]. Lee and coauthors reported VD decrease after successful silicone oil removal [23].

These studies analyzed retinal VD changes at three, six, and twelve months after the primary repair of RRD. After the examination of the literature related to this issue, we did not find any study that had a long term follow up in it. We were interested in the long term dynamics of retinal VD changes in eyes of RRD, therefore we divided our patients into three groups according to the elapsed time after RRD surgery: six months to one year, one year to two years, and more than two years (up to 10 years). OCT-A showed a significant reduction of VD in the superficial and deep regions of the macula after RRD surgery. We found that the difference in VD between the fellow eyes changes with time. The deep and parafoveal region is more affected in the earlier postoperative time. The superficial, the foveal region, and the extent of the non-flow area are more involved after a longer postoperative time. Our findings provide an insight into the long-term conformation of macular vasculature after the primary repair of RRD.

Our study has some limitations originating from its cross-sectional design. No prospective follow-up with OCT-A was possible to perform due to the long follow-up period, since the instrument was not available many years ago. Relying on OCT-A data it is very complicated to differentiate the exact pathomechanism of macular vascular changes. It is not determinable to state, that the changes are related to the retinal detachment itself or the surgical procedure. It has to be considered, that RD can have several different stages, and the surgical technique is variable, including endotamponade material. In a recent review Christou summarizes, that the exact macular vascular density changes are still not clear due to the diversity of RRD and surgical techniques [24].

Long time prospective OCT-A studies could provide evidence on that question, however gaining good quality OCT-A image from a detached retina is very challenging, and in the very early postoperative time, the endotamponade limits OCT-A image quality.

## Supporting information

S1 File (PDF)

## References

[1] Pastor JC, Fernández I, Rodríguez de la Rúa E, Coco R, Sanabria-Ruiz Colmenares MR, Sánchez-Chicharro D, et al. Surgical outcomes for primary rhegmatogenous retinal detachments in phakic and pseudophakic patients: the Retina 1 Project—report 2. Br J Ophthalmol. 2008;92(3):378–82. doi: 10.1136/bjo.2007.129437 . 18303159

[2] Borowicz D, Nowomiejska K, Nowakowska D, Brzozowska A, Toro MD, Avitabile T, et al. Functional and morphological results of treatment of macula-on and macula-off rhegmatogenous retinal detachment with pars plana vitrectomy and sulfur hexafluoride gas tamponade. BMC Ophthalmol. 2019;19(1):118. doi: 10.1186/s12886-019-1120-3; PMCID: PMC6534838. 31126280 PMC6534838

[3] Balogh A, Milibák T, Szabó V, Nagy ZZ, Resch MD. Position of macula lutea and presence of proliferative vitreoretinopathy affect vitreous cytokine expression in rhegmatogenous retinal detachment. PLoS One. 2020;15(6):e0234525. doi: 10.1371/journal.pone.0234525; PMCID: PMC7295219. 32542038 PMC7295219

[4] Coppola M, Marchese A, Cicinelli MV, Rabiolo A, Giuffrè C, Gomarasca S, et al. Macular optical coherence tomography findings after vitreoretinal surgery for rhegmatogenous retinal detachment. Eur J Ophthalmol. 2020;30(4):805–816. doi: 10.1177/1120672120911334 . 32174150

[5] Kobayashi M, Iwase T, Yamamoto K, Ra E, Murotani K, Matsui S, et al. Association Between Photoreceptor Regeneration and Visual Acuity Following Surgery for Rhegmatogenous Retinal Detachment. Invest Ophthalmol Vis Sci. 2016;57(3):889–98. doi: 10.1167/iovs.15-18403 . 26943151

[6] Park DH, Choi KS, Sun HJ, Lee SJ. FACTORS ASSOCIATED WITH VISUAL OUTCOME AFTER MACULA-OFF RHEGMATOGENOUS RETINAL DETACHMENT SURGERY. Retina. 2018;38(1):137–147. doi: 10.1097/IAE.0000000000001512 . 28099315

[7] Tani P, Robertson DM, Langworthy A. Prognosis for central vision and anatomic reattachment in rhegmatogenous retinal detachment with macula detached. Am J Ophthalmol. 1981;92(5):611–20. doi: 10.1016/s0002-9394(14)74651-3 . 7304687

[8] Woo JM, Yoon YS, Woo JE, Min JK. Foveal Avascular Zone Area Changes Analyzed Using OCT Angiography after Successful Rhegmatogenous Retinal Detachment Repair. Curr Eye Res. 2018;43(5):674–678. doi: 10.1080/02713683.2018.1437922 . 29451996

[9] Wang H, Xu X, Sun X, Ma Y, Sun T. Macular perfusion changes assessed with optical coherence tomography angiography after vitrectomy for rhegmatogenous retinal detachment. Graefes Arch Clin Exp Ophthalmol. 2019;257(4):733–740. doi: 10.1007/s00417-019-04273-7 . 30796563

[10] Tsen CL, Sheu SJ, Chen SC, Wu TT. Imaging analysis with optical coherence tomography angiography after primary repair of macula-off rhegmatogenous retinal detachment. Graefes Arch Clin Exp Ophthalmol. 2019;257(9):1847–1855. doi: 10.1007/s00417-019-04381-4 . 31177300

[11] Agarwal A, Aggarwal K, Akella M, Agrawal R, Khandelwal N, Bansal R, et al. FRACTAL DIMENSION AND OPTICAL COHERENCE TOMOGRAPHY ANGIOGRAPHY FEATURES OF THE CENTRAL MACULA AFTER REPAIR OF RHEGMATOGENOUS RETINAL DETACHMENTS. Retina. 2019;39(11):2167–2177. doi: 10.1097/IAE.0000000000002276 . 30080742

[12] Czakó C, István L, Ecsedy M, Récsán Z, Sándor G, Benyó F, et al. The effect of image quality on the reliability of OCT angiography measurements in patients with diabetes. Int J Retina Vitreous. 2019;5:46. doi: 10.1186/s40942-019-0197-4; PMCID: PMC6829984. 31709114 PMC6829984

[13] Karacorlu M, Sayman Muslubas I, Hocaoglu M, Arf S, Ersoz MG. Correlation between morphological changes and functional outcomes of recent-onset macula-off rhegmatogenous retinal detachment: prognostic factors in rhegmatogenous retinal detachment. Int Ophthalmol. 2018;38(3):1275–1283. doi: 10.1007/s10792-017-0591-6 . 28602014

[14] Sato T, Wakabayashi T, Shiraki N, Sakaguchi H. Retinal thickness in parafoveal subfields and visual acuity after vitrectomy for macula-off rhegmatogenous retinal detachment repair. Graefes Arch Clin Exp Ophthalmol. 2017;255(9):1737–1742. doi: 10.1007/s00417-017-3716-8 . 28639156

[15] Han KJ, Lee YH. Optical coherence tomography automated layer segmentation of macula after retinal detachment repair. PLoS One. 2018 May 7;13(5):e0197058. doi: 10.1371/journal.pone.0197058; PMCID: PMC5937759. 29734400 PMC5937759

[16] Lauermann JL, Woetzel AK, Treder M, Alnawaiseh M, Clemens CR, Eter N, et al. Prevalences of segmentation errors and motion artifacts in OCT-angiography differ among retinal diseases. Graefes Arch Clin Exp Ophthalmol. 2018 Oct;256(10):1807–1816. doi: 10.1007/s00417-018-4053-2 Epub 2018 Jul 7. . 29982897

[17] Lauermann JL, Treder M, Heiduschka P, Clemens CR, Eter N, Alten F. Impact of eye-tracking technology on OCT-angiography imaging quality in age-related macular degeneration. Graefes Arch Clin Exp Ophthalmol. 2017 Aug;255(8):1535–1542. doi: 10.1007/s00417-017-3684-z Epub 2017 May 4. . 28474129

[18] Lee JY, Kim JY, Lee SY, Jeong JH, Lee EK. Foveal Microvascular Structures in Eyes with Silicone Oil Tamponade for Rhegmatogenous Retinal Detachment: A Swept-source Optical Coherence Tomography Angiography Study. Sci Rep. 2020 Feb 13;10(1):2555. doi: 10.1038/s41598-020-59504-3; PMCID: PMC7018724. 32054939 PMC7018724

[19] Ng H, La Heij EC, Andrinopoulou ER, van Meurs JC, Vermeer KA. Smaller Foveal Avascular Zone in Deep Capillary Plexus Is Associated with Better Visual Acuity in Patients after Macula-off Retinal Detachment Surgery. Transl Vis Sci Technol. 2020 Sep 24;9(10):25. doi: 10.1167/tvst.9.10.25; PMCID: PMC7521173. 33024618 PMC7521173

[20] Bonfiglio V, Ortisi E, Scollo D, Reibaldi M, Russo A, Pizzo A, et al. Vascular changes after vitrectomy for rhegmatogenous retinal detachment: optical coherence tomography angiography study. Acta Ophthalmol. 2019 Nov 26. doi: 10.1111/aos.14315. 31773840

[21] Jiang J, Chen S, Jia YD, Li R, Zhou JX, Li RM. Evaluation of macular vessel density changes after vitrectomy with silicone oil tamponade in patients with rhegmatogenous retinal detachment. Int J Ophthalmol. 2021;14(6):881–886. doi: 10.18240/ijo.2021.06.14 ; PMCID: PMC8165621. 34150544 PMC8165621

[22] Fang W, Zhai J, Mao JB, Li HD, Qian ZB, Chen CQ, et al. A decrease in macular microvascular perfusion after retinal detachment repair with silicone oil. Int J Ophthalmol. 2021 Jun 18;14(6):875–880. doi: 10.18240/ijo.2021.06.13 ; PMCID: PMC8165614. 34150543 PMC8165614

[23] Lee JH, Park YG. Microvascular changes on optical coherence tomography angiography after rhegmatogenous retinal detachment vitrectomy with silicone tamponade. PLoS One. 2021;16(3):e0248433. doi: 10.1371/journal.pone.0248433; PMCID: PMC7954302. 33711059 PMC7954302

[24] Christou EE, Stavrakas P, Batsos G, Christodoulou E, Stefaniotou M. Association of OCT-A characteristics with postoperative visual acuity after rhegmatogenous retinal detachment surgery: a review of the literature. Int Ophthalmol. 2021;41(6):2283–2292. doi: 10.1007/s10792-021-01777-2 . 33745033

