# Supplementary material for: Association between retinal vessel density and postoperative time after primary repair of rhegmatogenous retinal detachment
Source: PLoS One. 2021 Oct 1;16(10):e0258126. doi: 10.1371/journal.pone.0258126 (PMC8486093; doi:10.1371/journal.pone.0258126)
Supplement: S1 File — (PDF) [file pone.0258126.s001.pdf]

Group	Postop time	Fovea thick.		SPF fovea		SPF parafovea		SPF whole		D fovea		D parafovea		D whole		SPF non-flow		FAZ	
	(months)	RRD	Control	RRD	Control	RRD	Control	RRD	Control	RRD	Control	RRD	Control	RRD	Control	RRD	Control	RRD	Control
1	6	299	249	19,70	15,70	40,70	51,90	39,50	47,10	31,40	31,10	46,90	52,50	44,20	50,60	0,446	0,519	0,308	0,342
1	6	311	323	25,80	29,90	45,70	45,70	43,30	44,30	43,00	40,00	48,00	44,60	48,00	42,20	0,253	0,202	0,095	0,086
1	6	295	289	8,70	17,20	24,40	44,80	24,40	41,90	20,80	31,40	43,90	52,20	40,80	50,30	0,729	0,458	0,366	0,317
1	8	264	294	26,40	28,10	38,40	51,90	38,50	47,40	43,60	46,80	48,10	53,50	46,60	53,30	0,257	0,216	0,116	0,140
1	9	297	278	26,30	19,70	46,30	51,90	45,00	41,40	40,50	36,00	45,10	48,10	43,00	46,50	0,277	0,504	0,145	0,186
1	9	219	248	12,50	17,80	37,20	51,90	35,80	35,40	31,10	44,60	53,90	58,80	51,40	56,00	0,584	0,547	0,180	0,169
1	9	213	248	21,80	32,60	49,40	51,90	47,10	49,50	33,10	40,30	50,50	49,70	48,00	47,10	0,424	0,256	0,262	0,207
1	11	248	249	12,10	13,20	49,00	44,70	44,40	40,20	27,60	31,60	50,00	51,70	46,30	47,60	0,565	0,594	0,359	0,338
1	12	298	275	23,30	24,40	47,90	52,80	46,10	50,50	35,50	40,00	47,10	55,50	45,30	53,60	0,416	0,412	0,202	0,227
1	12	294	262	30,20	25,80	51,40	51,90	49,00	49,40	41,40	39,80	48,50	55,00	46,70	53,10	0,280	0,318	0,177	0,203
2	13	413	244	28,30	16,00	N/A	47,20	36,80	44,70	40,20	24,60	N/A	53,70	30,60	51,60	0,350	0,494	N/A	0,414
2	13	281	266	16,40	19,60	43,50	56,80	41,50	53,30	33,40	32,80	62,40	53,10	58,60	51,60	0,509	0,432	0,321	0,263
2	13	250	239	13,60	8,20	36,90	33,90	35,80	30,70	24,30	24,90	51,50	55,40	49,30	51,10	0,575	0,658	0,363	0,297
2	14	611	297	35,80	33,20	39,50	44,10	39,90	43,60	31,90	44,70	37,40	43,50	36,80	42,60	0,165	0,307	0,123	0,135
2	16	231	255	15,00	18,50	40,00	51,50	38,10	48,00	21,90	31,70	52,00	53,80	49,10	50,60	0,553	0,460	0,387	0,298
2	16	278	274	26,40	26,10	46,80	46,20	44,40	44,10	41,10	43,10	50,40	54,30	48,40	52,30	0,330	0,322	0,111	0,143
2	16	218	231	6,70	6,50	41,90	48,40	38,80	44,60	20,60	15,80	54,70	50,40	50,00	45,90	0,714	0,728	0,507	0,542
2	17	472	277	28,50	22,60	41,10	41,30	39,00	38,70	43,80	40,70	44,50	50,60	44,40	48,10	0,253	0,314	0,052	0,066
2	18	292	267	15,90	23,10	30,60	47,30	29,20	45,50	31,90	35,00	44,50	48,40	42,50	47,90	0,526	0,295	0,212	0,191
2	21	327	282	19,90	13,70	42,80	40,50	41,10	38,90	36,20	35,50	43,00	55,70	41,50	53,00	0,297	0,608	0,147	0,190
3	25	229	247	16,90	19,80	31,70	41,60	31,20	39,80	26,30	34,80	54,70	52,30	48,60	48,80	0,632	0,453	0,298	0,269
3	34	260	266	21,80	23,20	39,70	39,30	38,70	36,30	37,10	46,30	50,60	50,40	48,20	48,10	0,360	0,380	0,209	0,093
3	46	293	289	33,10	29,80	51,30	47,40	49,80	45,10	50,70	47,80	55,60	52,40	54,80	51,30	0,193	0,214	0,114	0,064
3	56	274	251	5,10	25,60	25,20	51,80	23,50	48,90	29,50	40,20	32,40	55,00	33,00	53,70	1,606	0,350	0,055	0,238
3	70	279	268	26,90	26,70	44,80	47,60	43,00	45,60	39,20	42,40	45,50	55,30	44,40	54,10	0,339	0,318	0,182	0,189
3	72	217	234	9,40	10,00	45,10	44,00	41,30	41,00	20,70	29,80	42,50	60,50	39,50	57,30	0,642	0,626	0,441	0,378
3	77	264	260	13,40	10,40	39,00	42,90	37,30	38,60	25,20	24,30	51,90	48,30	50,10	44,50	0,546	0,641	0,420	0,319
3	77	282	262	16,80	19,80	34,70	50,20	34,80	47,90	42,10	34,30	66,20	50,50	62,00	47,10	0,474	0,423	0,247	0,274
3	79	231	316	10,90	22,70	38,90	36,40	35,40	34,90	29,70	32,40	46,90	47,60	44,60	45,50	0,624	0,422	0,298	0,310
3	79	245	324	17,10	27,80	35,50	50,90	34,20	48,60	33,10	36,90	49,20	44,40	45,70	43,40	0,419	0,384	0,223	0,328
3	98	205	265	18,00	21,50	46,50	54,50	43,10	51,00	24,50	35,70	44,10	54,30	41,20	52,00	0,444	0,415	0,337	0,232
3	110	245	330	18,50	32,40	35,00	49,60	33,80	47,70	30,80	40,90	45,90	47,00	44,00	46,90	0,665	0,251	0,220	0,171
3	126	279	266	18,20	23,80	29,20	51,30	29,30	48,20	31,00	39,40	47,30	56,10	45,70	54,50	0,443	0,291	0,170	0,196
